# Graphene Nanomaterials-Based Radio-Frequency/Microwave Biosensors for Biomaterials Detection

**DOI:** 10.3390/ma12060952

**Published:** 2019-03-21

**Authors:** Hee-Jo Lee, Jong-Gwan Yook

**Affiliations:** 1 Department of Physics Education, College of Education, Daegu University, Gyeongsan, Gyeongbuk 38453, Korea; 2 School of Electrical and Electronic Engineering, College of Engineering, Yonsei University, Seoul 03722, Korea

**Keywords:** graphene nanomaterials, radio-frequency, microwave, biosensor, wireless biomedicine

## Abstract

In this paper, the advances in radio-frequency (RF)/microwave biosensors based on graphene nanomaterials including graphene, graphene oxide (GO), and reduced graphene oxide (rGO) are reviewed. From a few frontier studies, recently developed graphene nanomaterials-based RF/microwave biosensors are examined in-depth and discussed. Finally, the prospects and challenges of the next-generation RF/microwave biosensors for wireless biomedical applications are proposed.

## 1. Introduction

**Figure 1:**
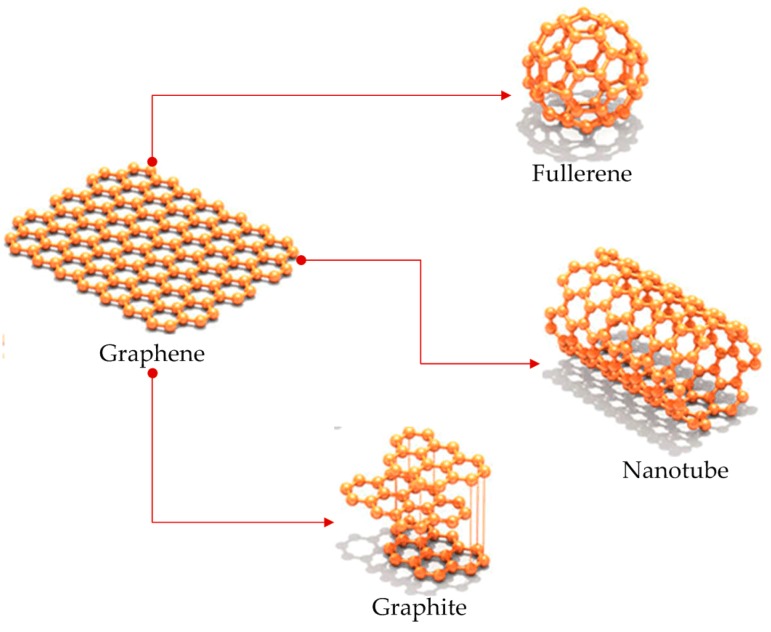
Allotropes of carbon: fullerene, nanotube, graphene, and graphite (reprinted with permission from [20]).

Graphene is a carbon allotrope consisting of an atomically thin two-dimensional (2D) hexagonal lattice [1]. This thin material can be regarded as the fundamental building block for the other carbon allotropes [2], i.e., three-dimensional (3D) graphite, one-dimensional (1D) carbon nanotubes, and zero-dimensional (0D) fullerenes [3], as shown in Figure 1.

**Figure 2:**
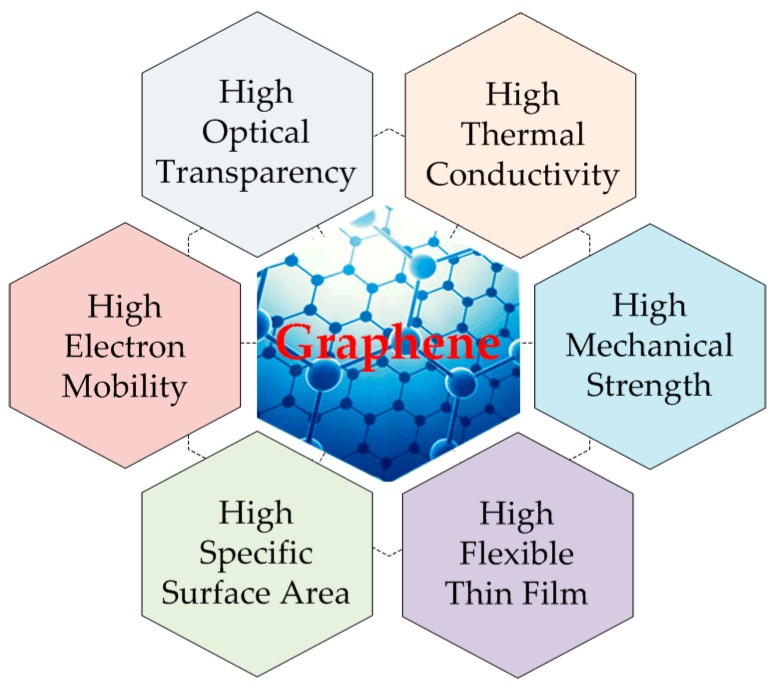
Excellent physical properties of pristine graphene.

In terms of physical properties, a pristine graphene without defects and impurities has a number of superior qualities such as high electronic mobility (~250,000 cm^2^·V^−1^·s^−1^) [4], high optical transparency (~97.7%) [5], high electrical, and thermal conductivity (above 3000 W·m^−1^·K^−1^) [6]. Pristine graphene also has high mechanical stiffness, strength (~130 GPa), and elasticity (~1.0 Tpa) [7], as shown in Figure 2. Owing to these excellent properties, graphene has become a good candidate material for diverse applications, particularly in graphene-based electronics such as flexible and transparent touch screens and organic light-emitting diodes [8]. In addition, research and development of graphene has been examined for enhancing the performance of the conventional radio-frequency (RF)/microwave devices and circuits such as graphene field-effect transistors with a cut-off frequency of 300 GHz [9], graphene antennas for radio-frequency identification (RFID) [10], microstrip attenuators operating in the frequency band from 1 GHz to 20 GHz [11], and graphene composites in electromagnetic shielding [12].

Meanwhile, graphene has excellent merits for biomedical applications, e.g., drug delivery and tissue engineering, because of its large surface area [13], chemical purity [14], easy functionalization [15], and many others. The unique electrical and mechanical properties of graphene [16] such as its ultimate thinness, conductivity, and strength makes it a robust support for imaging biomaterials in transmission electron microscopy [17]. In addition, graphene treated with biological functionalization might exhibit a rapid and ultra-sensitive response that can detect specific biomolecules such as glucose, cholesterol, hemoglobin, and DNA [18]. However, there is still room for a fundamental study to be carried out in order to understand the interaction between these biomolecules and pristine graphene [19].

In particular, graphene oxide (GO) and reduced graphene oxide (rGO) have received much attention as excellent nanomaterials for drug delivery due to their structural and surface properties that are well suited for biomedical applications [21,22]. In particular, the single-layer 2D structure of both GO and rGO provide an ultra-high specific surface area and delocalized π electrons on their surfaces that allows for the highly efficient loading of hydrophobic anti-cancer drugs through hydrophobic interactions and π–π stacking. In addition, they provide different oxygen-containing functional groups for easy biological functionalization [23,24,25,26]. Despite the unique and excellent properties of graphene nanomaterials for biomedical applications, graphene nanomaterials-based biomedical applications in the RF/microwave field are in their infancy. Fortunately, a few frontier research groups have recently opened the possibility of utilizing a graphene-based RFID system for wireless biomedical application [27,28].

This review outlines and highlights the recent trends and advances of RF/microwave biosensors based on graphene nanomaterials with their particular focus on the following: (1) graphene nanomaterials as biosensing materials, (2) the basic concept of RF/microwave biosensors, (3) RF/microwave devices and circuits based on graphene nanomaterials, (4) RF/microwave biosensors based on graphene nanomaterials, (5) the recent advancements and trends, and finally (6) the conclusions and prospects.

## 2. Graphene Nanomaterials for Biomedical Applications

In biomedical applications, all graphene derivatives are useful and extensively applied as components or as ingredients for biosensors, real-time bioimaging, cancer diagnosis and treatment, catalysis, and water purification [29,30,31,32,33,34,35]. These graphene derivatives are GO, rGO, few-layer graphene, wrinkled graphene, rGO hybridized with nanoparticles, fluorographene, hydrogenated graphene, and nano-sized GO. However, this review focuses on the graphene nanomaterials of graphene, GO, and rGO because these materials are mainly used in recently developed RF/microwave biosensors. The structure and merits of these graphene nanomaterials for biosensing are considered because these materials are widely used from zero-frequency (DC) to high-frequency (RF/microwave) bioelectronics, as summarized in Table 1.

As previously mentioned, graphene is a very thin nanomaterial with a single-layer of sp^2^ hybridized carbon atoms organized in the form of a honeycomb lattice. Preparation or synthesis methods have been well reported such as mechanical exfoliation from graphite, chemical vapor deposition (CVD), and molecular beam epitaxy (MBE). For GO, its synthesis procedures introduce a wide variety of oxygen-containing functional groups, such as carboxyl, hydroxyl, carbonyl, ethoxy, and epoxy, on both planes of the GO sheets, thereby increasing its stability in aqueous solutions. This also provides easy functionalization and derivatization of these materials through both covalent and non-covalent modifications with different biocompatible polymers such as polyethylene glycol and chitosan, and conjugation with targeting moieties, including peptides and antibodies, to develop bio-functionalized nanocomposite systems with improved biological properties [36,37]. rGO is much more efficient than GO because of the presence of more defects and greater aromaticity in its structure that are introduced during the reduction of GO using a variety of chemical, thermal, and electrochemical methods. 

**Figure 3:**
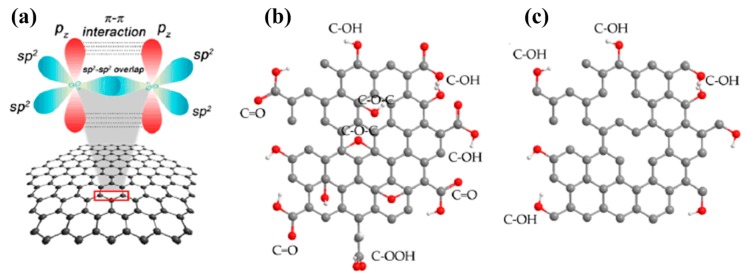
Structures of graphene nanomaterials: (**a**) Graphene with sp^2^-hybridized carbon atoms; (**b**) Chemically modified graphene including graphene oxide (GO); (**c**) reduced graphene oxide (rGO) (reprinted with permission from [41]).

For this reason, the excellent physicochemical and biocompatible properties of these graphene nanomaterials have created a wide variety of applications in the field of biomedicine. These are mostly in cancer research, ranging from nanocarriers and photo agents for drug/gene delivery to cancer cells as well as for photothermal and photodynamic therapies of cancer, both in vitro and in vivo, to its use as bioimaging agents for developing multifunctional theranostic platforms for carrying out more efficient detection and treatment of cancers [38]. As shown in Table 1, synthesis methods have prepared graphene nanomaterials for possible and further enhancements in this material, particularly in the biomedical field because of the incorporation of new features in these derivatives, as shown in Figure 3 [39,40,41]. Furthermore, valuable insights are reported on the differences in biological behavior between large and small sheet, single-layer, few-layer and multi-layer graphene samples [30], while the amount of oxygen can also provoke underside effects [42]. In addition, there is improvement of toxicity for biocompatibility of graphene derivatives [43].

## 3. Concept of Radio-Frequency/Microwave Biosensor

**Figure 4:**
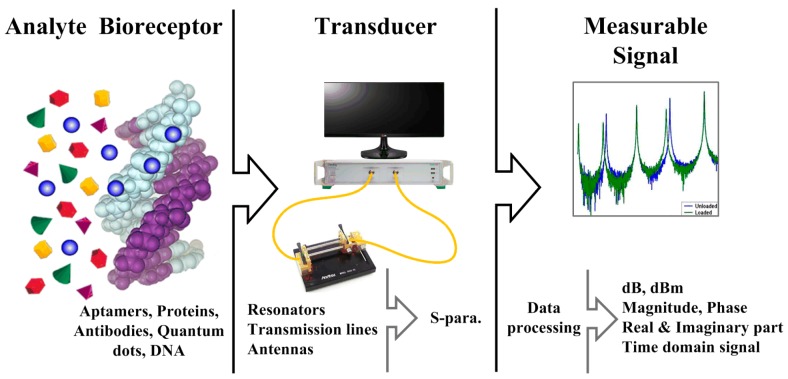
Basic concept of radio-frequency (RF)/microwave biosensors (reprinted with permission from [53]).

Generally, a biosensor is an analytical device that combines a biological sensing element with a transducer [51]. As shown in Figure 4, a biosensor commonly consists of three parts: biosensing, transducing, and signal processing [52]. First, the sensing part can be incorporated with sensitive biological elements such as aptamers, proteins, antibodies, and nucleic acids. These biological components are immobilized onto the electrode surface for the detection of specific analytes. Here, graphene and GO can be introduced as a matrix electrode and be immobilized for the detection of specific biomaterials. For an RF/microwave biosensing scheme, the patterned graphene and a GO flake are located at the gap between the electrodes or are connected as a part of circuits, such as resonator, transmission line, and antenna and these nanomaterials are then functionalized for specific binding, likewise antigen-antibody. Finally, the biological sensing information on graphene nanomaterials is converted to an observable signal via an RF/microwave measurement system. Here, the measurable signal is generally proportional to the concentration of a specific analyte [52]. 

### 3.1. Biological Functionalization Based on Graphene-Based Nanomaterials

In the previous session, the biological effects of graphene nanomaterials using diverse synthesis methods have been exploited for biomedical applications because of a large surface area that is capable of being immobilized on its surface and has easy-functionalization. Indeed, there are many possible approaches in the biomedical applications such as bioelectronics [54], tissue engineering [55,56,57], drug delivery [56,58], antibacterial materials development [59,60], biosensing [61,62], gene delivery [63], and cancer treatment [64], to engineer receptors for targeting biomaterials through various biological functionalization [65]. 

**Figure 5:**
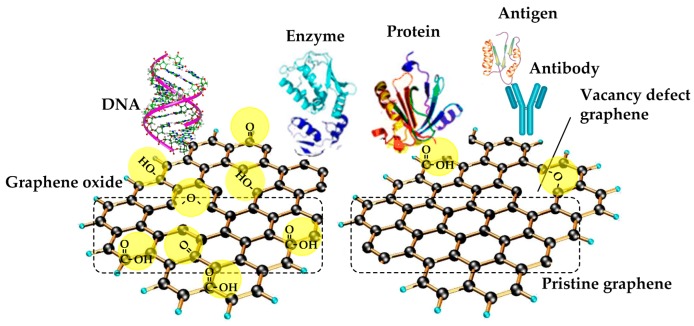
Biological functionalization of graphene nanomaterials: pristine graphene and GO (reprinted with permission from [41]).

In particular, as pristine graphene has a thin film of infinite size with no imperfections which provides a large number of chemically active sites for charge-biomolecular interactions due to the large surface area, however these also lead to an enhanced sensitivity to the target biomolecules of very low concentrations and improved selectivity [41]. For instance, Figure 5 presents the possible interactions on the pristine graphene and GO for detecting specific biological systems such as antigen-antibody, protein, enzyme, and DNA [66,67,68]. 

### 3.2. Radio-Frequency/Microwave Devices and Circuits Based on Graphene Nanomaterials

Over the past decade, the feasibility of graphene in RF/microwave electronics was investigated owing to its excellent physical properties. As shown in Table 2, the studies of graphene nanomaterials as RF/microwave devices and circuits such as resonators, transmission lines, antennas, and interconnects have been carried out. In particular, the graphene-based resonator has been widely examined on the feasibility as an RF/microwave biosensing device because of its easy characterization and fast discrimination of the biomolecular detection [69,70]. In the case of GO, this has been frequently used as a type of biological matrix at the gap between RF/microwave electrodes or conventional devices and circuits, e.g., interdigitated capacitor and transmission line, and so on [71,72]. In the case of RF/microwave, as frequency increases, the skip depth is very thin so that most current flows on the circuit surface. For this reason, although the depth of surface current is larger than graphene thickness, i.e., a few nanometers, graphene nanomaterials can, potentially, be used as sensing materials for the highly sensitive RF/microwave biosensor to detect various biomaterials via biological functionalization.

### 3.3. Radio-Frequency/Microwave Sensing Parameters

In RF/microwave measurement system, the measured data are primarily related to the S-parameters (or S-matrix), which is defined as the ratio of the output power (voltage) to the input power (voltage) in the frequency domain. In particular, the S-parameters for the two-part network are expressed as

(1) $$\left[S\right]=\left[\begin{array}{cc} S_{11} & S_{12}\\ S_{21} & S_{22} \end{array}\right]$$

where S_11_ (or S_22_) is the reflection coefficient of the input (or output) port, and S_21_ (or S_12_) is the transmission coefficient of the output port to the input port (or output port to the input port), respectively. The measured S-parameters can be converted to many other parameters such as admittance (Y), impedance (Z), hybrid (H), transfer (T), and ABCD matrix [84]. Owing to the relationship between these parameters, the observable data can allow diverse analysis and processing such as dB, dBm, magnitude, phase, real and imaginary, and time domain signal, for specific biosensing events.

## 4. Radio-Frequency/Microwave Biosensors Based on Graphene Materials

### 4.1. Case Studies: Radio-Frequency/Microwave Biosensors Based on Graphene Oxide

GO has received considerable interests as a sensitive material for biosensing because electron transfer from the biomolecular binding mainly occurs at the defects or edges of GO structure. In addition, owing to the opposite properties of a hydrophobic and hydrophilic functional group on GO, this material exhibits good biocompatibility, high affinity for specific biomolecules. Hence, these properties of GO can provide many opportunities for the various approaches of biosensing platforms, including biosensors based on fluorescence resonance energy transfer, laser desorption/ionization mass spectrometry, surface-enhanced Raman spectroscopy, and electrochemical detection [85]. 

**Figure 6:**
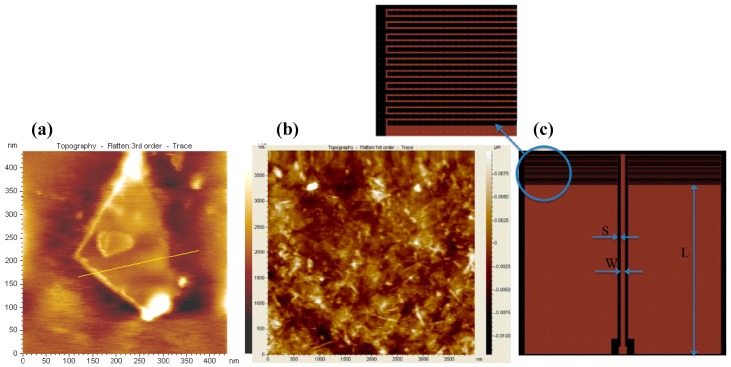
RF/microwave biosensor based on coplanar waveguide (CPW) line with meander resonator: atomic force microscope (AFM) image of GO flake (**a**) diluted 1:100 ratio; (**b**) concentrated samples; (**c**) CPW line with meander resonator, where *L* and *W* are the width and the length of the signal line, respectively and *S* is the spacing between the signal line and ground (reprinted with permission from [87]).

Figure 6 presents the biosensing scheme based on the microwave impedance for detecting DNA on GO sheets. The sensing scheme is a type of coplanar waveguide (CPW) line with meander resonator fabricated on the wafer. In this biosensing scheme, GO sheets (~1.3 nm-thickness) were coated on the resonator part. Surface topology of GO flake was examined by atomic force microscope (AFM). For specifically biological functionalization, a medium molecule weight chitosan was dropped on the GO-coated region, and then calf thymus DNA (1 μg/mL) was also dropped on the chitosan-linked GO matrix [86]. Chitosan was used to adhere to the DNA on the GOs. In this work, the impedance characterization of the graphene nano-platelets attached to the sensing platform was carried out at up to 10 GHz. A remarkable change in impedance was observed. As a result, this work demonstrated that the RF/microwave impedance of GO materials could be used as sensing vehicles for future biological-sensing and chemical-sensing [87].

**Figure 7:**
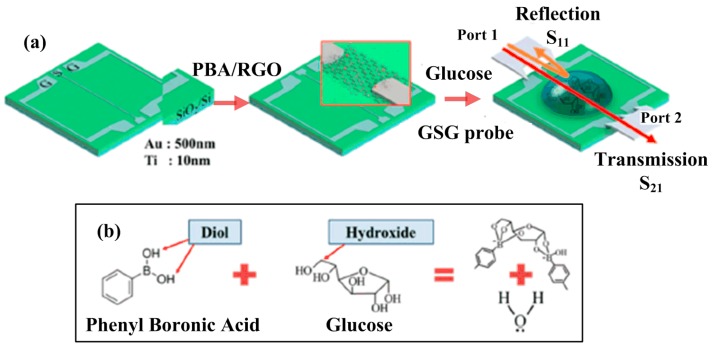
RF/microwave biosensor based on rGO for sensing of glucose molecule: (**a**) Schematic diagram of glucose sensor; (**b**) Glucose binding to the phenyl butyric acid (PBA) linker (reprinted with permission from [89]).

Figure 7 also shows the sensing scheme based on RF/microwave interconnects circuit for the detection of glucose. Here, rGO was functionalized with a phenyl butyric acid (PBA) linker to be able to detect the glucose molecules. Here, glucose is a critical molecule required for the normal growth of a cell, and the management of diabetes mellitus requires continuous monitoring of the blood sugar levels [88]. In this study, the glucose sensor showed the linear characteristics to the RF signal change with the concentration of glucose solution. From the experimental results, the rGO-based biosensor was detectable in small glucose concentration (1–4 mM) by utilizing transmission line parameters, i.e., resistance (R), inductance (L), conductance (G), and capacitance (C), as summarized in Table 3. In particular, this work demonstrated that the *R* component revealed the sensing parameter for effectively detecting the glucose level with stable linearity and small fluctuation [89].

### 4.2. Case Studies: RF/Microwave Biosensors Based on Graphene

One of the excellent physical properties in graphene is a transparent and conducting material with low cost and low environmental impact. This is an ideal material for the integrant of biosensing devices in a range of transduction modes, from electrochemical transduction to optical transduction [90].

**Figure 8:**
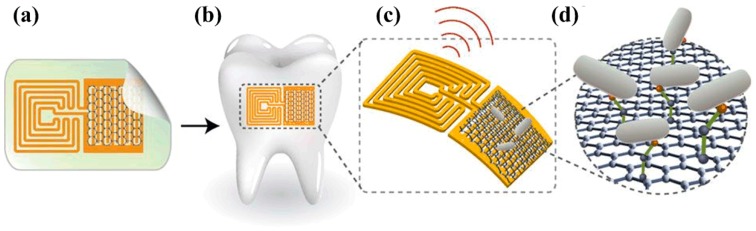
Graphene-based RF/microwave biosensor for the detection of bacteria: (**a**) Graphene patterned onto bioresorbable silk and contacted with wireless coil; (**b**) bio-transfer of the nano-sensing architecture onto the surface of a tooth; (**c**) magnified schematic diagram of the sensing element; (**d**) binding of pathogenic bacteria by peptides self-assembled on graphene (reprinted with permission from [27]).

Figure 8 presents the biosensing scheme base on an RFID sensor system with a graphene pattern for a few bacteria detection on tooth enamel. First of all, to detect bacteria specifically, biological functionalization of graphene was treated with bifunctional peptides for efficient recognition of pathogenic bacteria, e.g., *Odorrana grahami*, *H. pylori*, *E. coli*, and *S. aureus* [91]. These bacteria were recognized by utilizing specific peptide self-assembled on graphene. In this biosensing scheme, graphene was patterned on water-solution silk, and it was possible to recognize the remote pathogenic bacteria by utilizing an inductor (*L*)-capacitor (*C*) circuit, i.e., a resonant circuit for selecting a specific frequency. Also, this study demonstrated integration onto a tooth for specific bacteria detection in saliva via wireless circuitry [27].

## 5. Recent Research Trends: RF/Microwave Biosensors Based on Graphene Nanomaterials for Wireless Biomedical Applications

Recent advances in integrated biosensing platforms associated with remote sensing via RF/microwave wireless systems have focused on design and architecture of point-of-care (POC) diagnosis, attracting considerable interest in the biomedical applications. In particular, POC has significant diagnosis possibilities for use in the continuous and real-time monitoring of human metabolites as well as cancer biomarkers [92]. In addition, flexible and stretchable-integrated biosensors can directly monitor metabolic changes on the human body and quantify the electrically fine signals generated by specific bodily fluids. As a result, from this biosensing scheme, the wearable biosensors that can be attached intimately in the skin or tissue offer new opportunities for medical diagnostics and therapy. In recent years, there has been enormous progress in graphene-integrated wireless RF/microwave systems for real-time monitoring of metabolic change [93]. For example, a wireless smart soft contact lens system composed of reconfigurable capacitive sensor interface circuitry and wirelessly powered RFID addressable system for sensor control and data communication [94,95] was developed. In particular, monitoring for glucose and other biomarkers may become more sophisticated if the sensor is coated with graphene in this system.

## 6. Conclusions and Prospective

Recent advances in graphene nanomaterials such as synthesis techniques, electrical, thermal and mechanical analysis, surface treatment and device design have accelerated the development and application of graphene nanomaterials-based nanoelectronics as well as bioelectronics. In this review, we have examined the emerging advances of graphene nanomaterials-integrated biosensors including structures and merits of graphene nanomaterials and their biological functionalization in RF/microwave biomedical applications. From the developed RF/microwave biosensors, these biosensing schemes could be classified with passive RF/microwave devices and RF/microwave systems with graphene nanomaterials. Firstly, it was used as a biosensing scheme utilizing simple RF/microwave devices such as resonators and capacitors, with graphene nanomaterials like GO or rGO. In the case of latter, it was used as a biosensing scheme utilizing RF/microwave systems with graphene nanomaterials, e.g., graphene. These RF/microwave biosensors could be detectable of biomolecules, e.g., glucose, DNA, as well as bacteria, e.g., *S. aureus*, *E. coli* and so on, via bifunctional peptide. 

However, the research and development of these materials-based biosensing systems are in their infancy in the RF/microwave biomedical applications. This is because it is not only difficult to find the optimized frequency for biosensing, but devices and circuits also are dependent on the frequency. However, since there are great merits such as real-time, non-invasive, non-contact function, as a graphene nanomaterials-based RF/microwave biosensor, the biosensing scheme still needs to develop the robust biosensing platform integrated with wireless and flexible devices and circuits. In this case, there are also remains challenges how to find effective integration methods and how to secure stability for good performance of RF/microwave devices and systems with graphene nanomaterials. Before this challenge, the optimization of material fabrication and modification techniques to obtain large area, high quality, and uniform arrays will be essential for the highly sensitive and reproducible RF/microwave biosensors. Furthermore, the integration of graphene nanomaterials-based RF/microwave device needs to be optimized to minimize the entire device volume for portable, disposable and POC diagnosis and healthcare in the future.

## Figures and Tables

**Table 1:** Representative reparation and synthesis methods for graphene nanomaterials.

Graphene-Based Nanomaterials	Preparation and Synthesis Methods	Merits and Uses	Reference
Graphene	Mechanical exfoliation	Small-scale, high quality and basic research	[44]
	CVD ^1^	Large-scale, high quality and sensors	[45]
	Liquid exfoliation	Cost-effectiveness and transparent electrodes, sensors	[46]
	MBE ^2^	Easy integration into existing electronic procedures	[47]
GO	Hummers and Offeman	Less hazardous way	[48]
rGO	CRGO ^3^	Cost-effective and simple process using chemical solutions, high yield of graphene dispersion scalable for industry, highly stable collides	[49]
	TRGO ^4^	High surface area similar to pristine graphene	[50]
	ERGO ^5^	Material almost structure identical to pristine graphene	

Notes: ^1^ CVD: chemical vapor deposition, ^2^ MBE: molecular beam epitaxy, ^3^ CRGO: chemically reduced graphene oxide, ^4^ TRGO: thermally reduced graphene oxide, ^5^ ERGO: electrochemically reduced graphene oxide.

**Table 2:** RF/microwave devices and circuits of graphene nanomaterials.

Graphene-Based Materials	RF/Microwave Devices and Circuits	Reference
Graphene	Transmission lines	[73,74]
	Resonators	[75,76,77]
	Antennas	[78,79,80]
Graphite	Antennas	[81]
GO	Interconnects	[82,83]

**Table 3:** Types of rGO or GO-based RF/microwave biosensors.

RF/Microwave Device/Circuit Types	Sensing Parameters	Target Biomolecules	Limit of Detection	Reference
CPW line with meander resonator	*f*_r_^1^, R, X ^2^, ε_r_^3^	DNA	-	[87]
Interconnect	R, L, G, C	Glucose	1–4 mM	[89]

Notes: ^1^ *f*_r_: resonance frequency, ^2^ X: reactance, ^3^ ε_r_: relative permittivity.
